# Supplementary material for: Characterization of Crystal Microstructure Based on Small Angle X-ray Scattering (SAXS) Technique
Source: Molecules. 2020 Jan 21;25(3):443. doi: 10.3390/molecules25030443 (PMC7036759; doi:10.3390/molecules25030443)
Supplement: Supplementary file 1 [file molecules-25-00443-s001.pdf]

Supplementary Materials

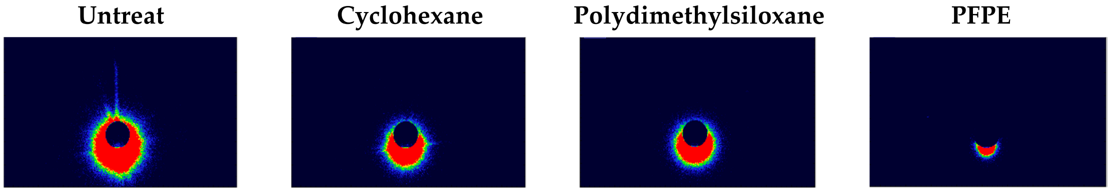

Figure S1. Selected SAXS patterns of HMX at room temperature taken at different matching solution as indicated on the graph.

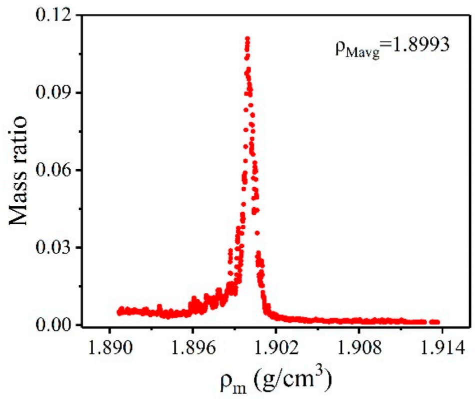

Figure S2. Mass density distribution of HMX.

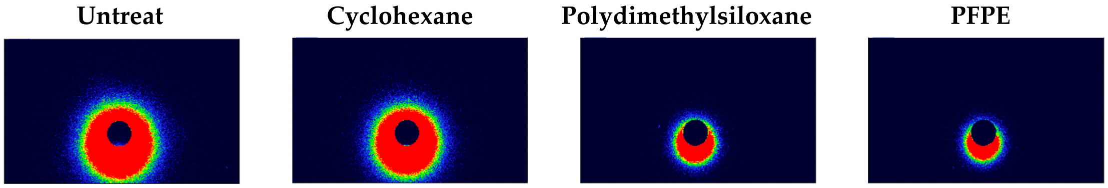

Figure S3. Selected SAXS patterns of CL-20-1 at room temperature taken at different matching solution as indicated on the graph.

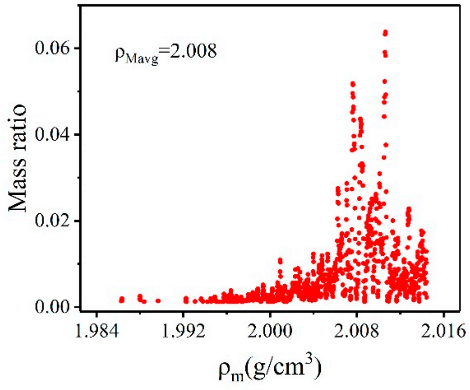

Figure S4. Mass density distribution of CL-20-1.

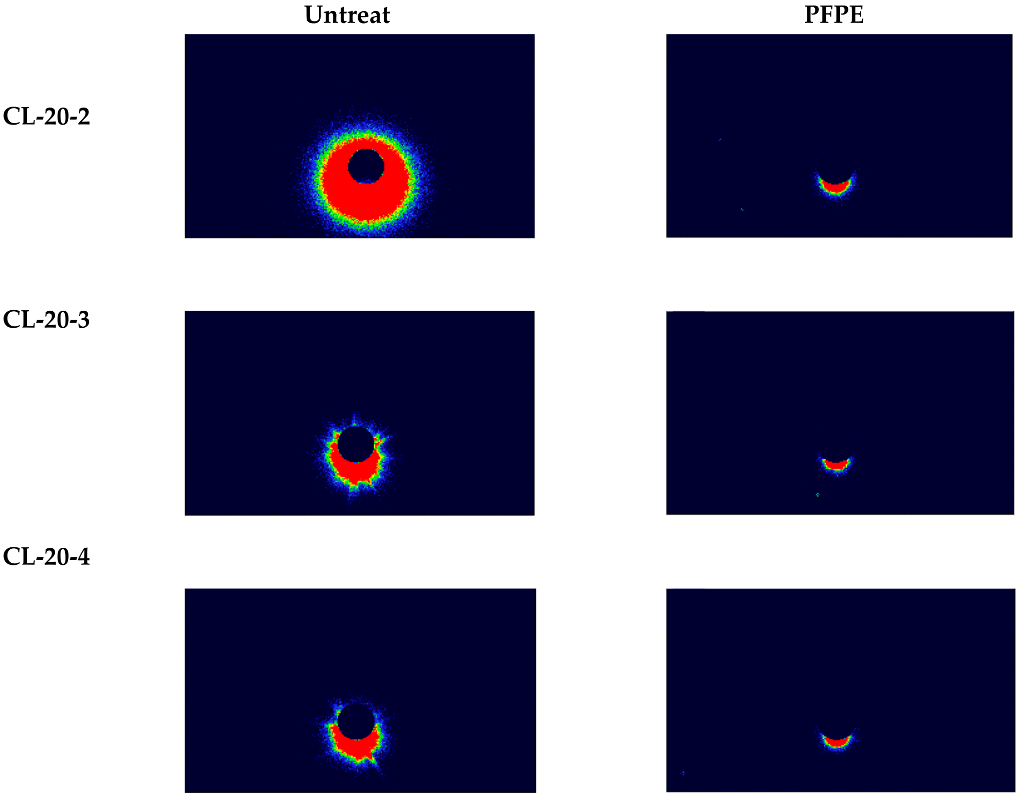

Figure S5. Selected SAXS patterns of CL-20-2、CL-20-3 and CL-20-4 at room temperature taken at different matching solution as indicated on the graph.

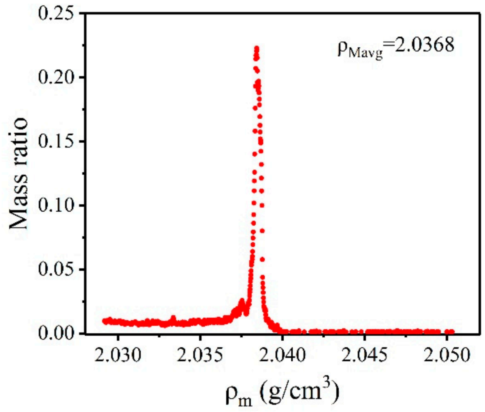

Figure S6. Mass density distribution of CL-20-2.

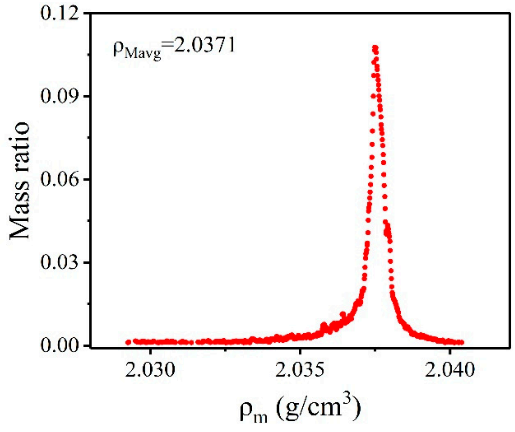

Figure S7. Mass density distribution of CL-20-3.

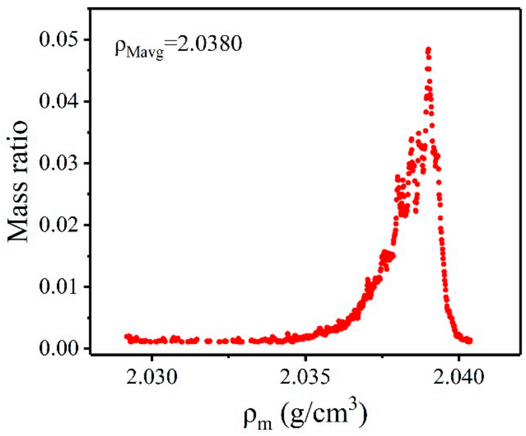

Figure S8. Mass density distribution of CL-20-4.

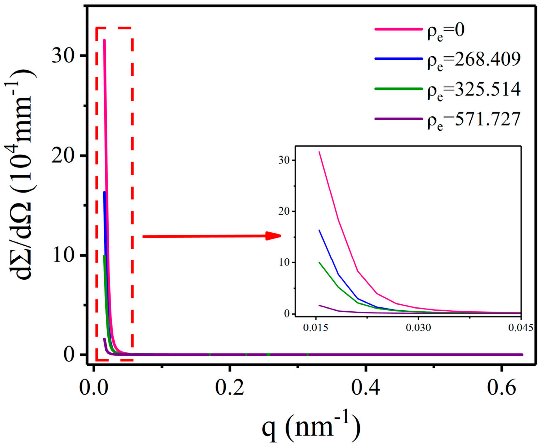

Figure S9. Representative SAXS curves of the HMX in different matching solutions.

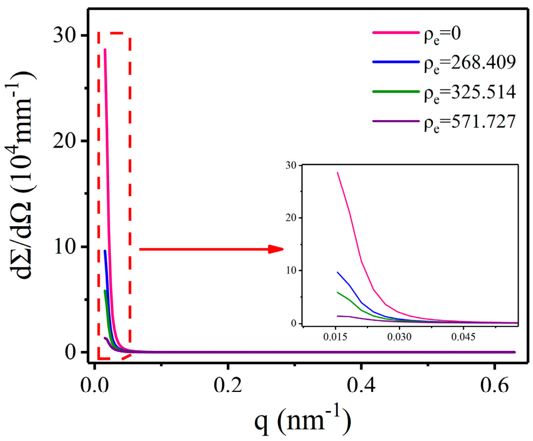

Figure S10. Representative SAXS curves of the CL-20-1 in different matching solutions.

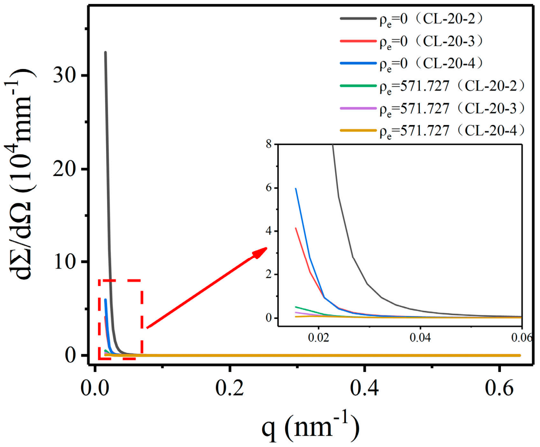

Figure S11. Representative SAXS curves of CL-20-2, CL-20-3 and CL-20-4 in different matching solutions.
